# Is self-sampling to test for high-risk papillomavirus an acceptable option among women who have been treated for high-grade cervical intraepithelial neoplasia?

**DOI:** 10.1371/journal.pone.0199038

**Published:** 2018-06-18

**Authors:** Sonia Andersson, Karen Belkić, Miriam Mints, Ellinor Östensson

**Affiliations:** 1 Department of Women’s and Children’s Health, Karolinska Institute, Stockholm, Sweden; 2 Department of Oncology-Pathology, Karolinska Institute, Stockholm, Sweden; 3 School of Community and Global Health, Claremont Graduate University, Claremont, California, United States of America; 4 Institute for Prevention Research, Keck School of Medicine, University of Southern California, Alhambra, California, United States of America; 5 Department of Medical Epidemiology and Biostatistics, Karolinska Institute, Stockholm, Sweden; Rudjer Boskovic Institute, CROATIA

## Abstract

**Objective:**

Self-sampling to test for high risk human papilloma virus (HPV) is becoming an increasingly important component of cervical cancer screening. The aim of this observational study is to examine how women treated for high-grade cervical intraepithelial neoplasia (CIN) view HPV self-sampling.

**Methods:**

Invited to participate in the present study were patients who had undergone treatment of high-grade CIN (grade 2 or higher) and were followed-up at 6-months at the Karolinska University Hospital, Stockholm. The participants were instructed as to how to perform HPV self-sampling. Thereafter, the participants completed a questionnaire about HPV self-sampling and other cervical cancer screening methods, as well as about self-perceived risk of cervical cancer without regular gynecologic follow-up and about specific knowledge regarding HPV, CIN and cervical cancer.

**Results:**

Altogether 479 women enrolled in this study. The participation rate was 96.6%. Nearly 75% of the participants stated they would consider performing the HPV self-sampling prior to their next gynecologic follow-up. Confidence in HPV self-sampling was a significant independent predictor of willingness to perform HPV self-sampling. However, confidence in HPV self-sampling was significantly lower than confidence in Papanicolaou smears and in HPV testing with samples collected by health professionals. Higher specific knowledge about HPV, CIN and cervical cancer was also a significant independent predictor of willingness to perform HPV self-sampling, as was having travelled longer distance to attend gynecologic follow-up. Participants with lower income and without completed university education expressed significantly higher confidence in HPV self-sampling and lower confidence in Papanicolaou smears than the other women.

**Conclusions:**

To the best of our knowledge, this is the first study to examine the views of women treated for high-grade CIN vis-à-vis HPV self-sampling. The latter is an acceptable option for the vast majority of this cohort of women.

## Introduction

Cervical cancer is the fourth most common cause of cancer mortality among women worldwide [1]. In 2012 alone it was estimated that 265,700 women succumbed to cervical cancer. Nearly all of these women were from less developed parts of the world [2], such that cervical cancer has been called “a disease of disparity”. These disparities in cervical cancer incidence and death are clearly seen in Europe [3]. The highest death rates from cervical cancer are reported in those European Union (EU) countries in which participation in screening programs is the lowest [3]. In most countries with cervical cancer screening programs, the majority of cervical cancers occur in women who have not been regularly screened [4]. Low income and low levels of education have frequently been associated with non-participation in cervical cancer screening [5–7], although there is some evidence to the contrary [8].

Screening programs and subsequent treatment of cervical dysplasia have successfully reduced cervical cancer mortality [9–11]. Since 1967, an invitational, population-based cervical cancer screening program has been on-going in Sweden. The cervical screening program entails a 3-year interval between negative screens for women aged 23–50 and 7 years for women aged 51–64 [12]. Cytological results are categorized according to the Bethesda nomenclature [13]. Primary screening for high-risk human papilloma virus (HPV) has been recently recommended for women aged 30–64, while cytology is the primary screening test for women aged 23–29 [12]. As of 2010, the participation rate in this Swedish program was reportedly 73%, with cervical cancer mortality rate the 9^th^ lowest in the EU [3, 14]. Despite these successes, in Sweden close to 500 women are diagnosed with cervical cancer each year and approximately 200 women per year die from this malignancy [15, 16].

Persistent infection with HPV has been identified as the key etiologic factor for cervical cancer. This finding is having a major impact upon cervical cancer screening and prevention strategies. Although frequently less specific than cytology-based examination, testing for HPV is reportedly more sensitive for detection of high-grade cervical intraepithelial neoplasia (CIN) [1, 9]. Overall, screening for high-risk HPV infection is found to be more effective than cytology via Papanicolaou (Pap) smear in reducing the incidence of cervical cancer [17]. In particular, it is now accepted to use HPV tests for triaging women with equivocal cervical cytology and for assessing risk of recurrence after treatment of high-grade CIN [17].

In addition to HPV testing on samples collected by health professionals, patients themselves can collect the samples for HPV testing. Self-sampling for HPV is one proposed approach for expanding access to cervical cancer screening. A meta-analytical comparison through mid-2013 of self-sampling versus clinician-collected samples indicated that the latter are more accurate [18]. In particular, the pooled sensitivity of self-collected samples was about 12% lower for detecting CIN2 or high-grade pathology. Nevertheless, the level of accuracy of self-sampling was considered to be sufficient for recommendation as an “additional strategy” among women who otherwise do not participate in screening [18]. Moreover, efforts to further improve the reliability of self-sampling are on-going [19–22]. Indeed, in a more recent study [23] with complete data for 136 patients attending colposcopy clinic, HPV testing using a clinically-validated HPV assessment was found to yield the same high level of accuracy on self-collected samples as on those collected by health professionals. This validation was based upon reproducibility and relative sensitivity and specificity compared to the two HPV DNA assays that were confirmed in large randomized trials and studies with follow-up of 8 years or more, as per Ref. [24].

Notwithstanding the earlier described limitations, self-collection of samples for HPV testing is becoming an increasingly important and accepted option for cervical cancer screening [5, 25–31]. In some settings, HPV self-sampling is preferred over other methods such as clinician-collected HPV tests as well as Pap smear [32]. Home-based HPV self-sampling may effectively provide an acceptable alternative for women who are otherwise unscreened [5, 33–35]. Among other considerations, the cost-effectiveness of repeated HPV self-sampling within the framework of an organized screening program has been underscored in a modeling study [36].

For women in whom high-grade CIN has been detected and treated, these issues are of special importance. This group of women needs more intense follow-up than the general population, but evidence-based guidelines for the most appropriate screening protocols are lacking [9, 37–39]. An estimated 15% of women treated for high-grade CIN will have a recurrence or residual disease, usually within 2 years post-treatment [37]. Among 3273 patients treated for high-grade CIN or adenocarcinoma in situ, a considerable 5-year risk was reported of recurrent disease. Thus, it was concluded “no subgroup of women achieved risk sufficiently low to return to 5-year routine screening” (p. S79), although women with negative post-therapeutic cotests were found to be at lowest risk [39].

On the other hand, a negative HPV test plus negative cytology at six months were found to be a reliable test of cure after three-year follow-up among 330 women with cervical dysplasia treated with the loop electrical excision procedure (LEEP) [10]. Our group carried out a study among ninety patients followed-up after treatment for CIN2+ with the loop excision electrosurgical procedure using a C-LETZ electrode, finding that all five cases of residual disease were predicted by high-risk HPV genotypes. Furthermore, the absence of persistent HPV infection was the most specific sign that there was no recurrent or residual high-grade disease [40]. We subsequently performed a follow-up investigation of 149 patients, finding that all cases of treatment failure among the women with high-grade disease were predicted by high-risk HPV [41]. Concordantly, a longitudinal study of 310 patients with CIN2+ from Italy [42] reveals that none of the 172 women with negative HPV 6 months after treatment had recurrent or residual CIN2+ during the 2-year surveillance period. Consequently, negative HPV was considered to a strong predictor of disease eradication. A longitudinal investigation of 435 patients treated for CIN2+ revealed that 3 consecutive negative cytologies or 2 negative results of co-testing at 6 and 24 months, had a similar 5-year risk of high-grade CIN compared to the general population of women undergoing cervical screening [37]. A systematic review and meta-analysis indicates that among women treated for high-grade CIN, the sensitivity of HPV testing was higher than cytology in detecting post-treatment disease. It was thus recommended that HPV testing be included in post-treatment follow-up [43].

As noted, self-sampling to test for HPV has been quite extensively studied in a number of settings throughout the world. This modality could be a helpful option for women with high grade CIN. However, to the best of our knowledge, there have not been any published papers examining self-sampling to test for HPV among women diagnosed and treated for high-grade CIN. In the present study we focus upon HPV self-sampling among this cohort of women, examining this in relation to other diagnostic modalities, self-perceived risk of cervical cancer and knowledge about HPV, CIN and cervical cancer. Sub-groups within this cohort for whom this option might be particularly helpful will be examined in more depth. The wider aim of these efforts is to optimize and tailor follow-up for the needs of these women at increased risk for developing cervical cancer. Vital to these efforts is to ensure adequate safety against recurrent/residual disease.

## Methods

### Study design, participants and setting

As described in Ref. [44], to be eligible for the present observational study, the patient would have been treated for CIN2+. The majority of the patients had been initially treated at the Karolinska University Hospital. Some of the patients had been initially treated at Danderyd or South General Hospital, the two other hospitals that treat women residing in Stockholm County. All the patients were treated by conization. The patients subsequently attended the first follow-up at the Karolinska University Hospital 6 months after treatment. The patients were identified by the Research Coordinator (EÖ), who arranged the six-month follow-up.

A printed invitation letter was sent by postal mail to each eligible patient about 5 months after treatment. Therein, a short summary of key facts about HPV and cervical cancer were included, together with information about how to contact the Attending Gynecologist and Principal Investigator (SA) and the Research Coordinator (EÖ). The study was presented as including a self-collection of a sample for HPV testing and completion of a questionnaire. The stated aim of the study was to better prevent cervical cancer. Full assurance was given of confidentiality and freedom to withdraw from the study at any time without any adverse consequences whatsoever.

Major efforts were made by the clinical research team to arrange convenient scheduling of the 6-month follow-up appointment for each patient. Thereby, all the patients who had been treated for high-grade CIN came to the follow-up examination.

The Research Coordinator (EÖ) met with each patient to explain the study procedure at the 6-month follow-up visit. An informed consent form was signed by all the invited patients. The options were agreement or declining to participate in the study. The Karolinska Ethics Committee (2006/1273-31, 2014/2034-32) approved the study protocol.

Since the questionnaire was in Swedish, Swedish language proficiency was a requirement for participation in the questionnaire portion of the study. There were 480 patients who were eligible to participate in the questionnaire portion of the study. Altogether, 479 of these patients signed the informed consent agreeing to participate and thereby were included in this study. All but six of the patients included in this study were treated for CIN for the first time.

There were also sixteen patients who were treated for high-grade CIN and attended 6-month follow-up and who would otherwise have been eligible to participate. However, these sixteen patients were not fluent in Swedish, and thus did not receive the questionnaire. Including the latter sixteen non-participating patients, the participation rate was 96.6%. These seventeen non-participants were of a mean age of 42 years (standard deviation 7.8 years).

### Presentation and performance of the HPV self-sampling

At the clinical care site, and before the gynecologic examination, each study participant was given a written description of how to use the self-sampling kit (Qvintip Approvix AB Uppsala, Sweden) device for collection of the vaginal fluid specimens, as well as instructions for taking urine samples (with first void urine in a urine cup). The self-collection was performed at the restroom reserved for patients at the clinical care site, with the urine sample collected first. The samples were analysed for HPV with the Abbott RealTime high-risk HPV assay (Abbot GmbH & Co.KG, Westbaden Germany). After completing self-collection, the patient gave the samples to the Research Coordinator (EÖ) for further handling, according to the study protocol. The gynecological examination including colposcopy was performed by gynecologists with special expertise in colposcopy (SA, MM) with cervical scrape samples collected from each woman. All samples collected were analyzed for HPV using a clinically validated real-time polymerase chain reaction (PCR)-based test for the detection of HPV DNA. Close agreement of HPV results between self-collected vaginal and urine samples in comparison to physician-collected cervical reference material was found [45].

### Questionnaire

After the self-sampling in the restroom at the care site and before completion of the gynecological examination, each participant was given a printed questionnaire (S1). This was completed at the clinical care site, with full anonymity guaranteed, as per the consent form. Socio-demographic queries were posed in the first part of the questionnaire. Inquiries about incurred expenses, travel time and arrangements needed to attend the gynecologic examination were posed in part 2. Part 3 surveyed knowledge about CIN, HPV, and attendant risk of cervical cancer and other malignancies. These results were analysed in detail in Ref. [44]. These knowledge-related questions were akin to those used in our earlier study conducted among a broader group, namely, among women attending cervical cancer screening in Stockholm [46]. In our previous study [44], factor analysis was employed to develop a Specific Knowledge scale, used herein, with the following 6 of the 14 knowledge queries:

- Human papilloma virus (HPV) is sexually transmitted,
- HPV can be asymptomatic,
- HPV can persist leading to cell changes in the cervix,
- Cell changes in the cervix over a long period of time can lead to cervical cancer,
- Vaccination can protect against cell changes in the cervix and cervical cancer,
- Gynecologic screening is important even if vaccinated against HPV.

Included in part 4 was a rating scale from 10 to 1 (10 highest, 1 lowest), on how the participant views her own risk of developing cervical cancer if she does not receive regular gynecologic follow-up.

The final portion of the questionnaire concerned the HPV self-sampling. The patient was asked whether the self-sampling was easy for her to carry out, and if not, to state the reason(s) why not, as an open-ended question, whether the self-sampling instructions were clear, and if not, what was missing, as an open-ended question. The participant was then asked to rate on a scale from 10 to 1, (10 highest and 1 lowest), her confidence that cervical cell changes would be found, such that she would be protected from developing cervical cancer. The same query and rating scale were then presented for HPV tests from self-collected samples, and for Pap smear performed by health professionals. Thereafter, the participant was asked whether she could see herself performing HPV self-sampling at home before coming to the next gynecologic follow-up. Open queries ended the questionnaire: reasons for or against carrying out the self-sampling.

### Analysis of the data

Univariate analysis was thoroughly performed on available data from the study (S2). (Note questions F101 (age in years) and F102 (municipality) are not included in the data set to protect the patients’ confidentiality). Therein, the distribution of all continuous and semi-continuous variables was evaluated by inspection, as well as by examining skewness and kurtosis. When the continuous or semi-continuous variables had skewness as well as kurtosis less than one, and appeared to be normally distributed, parametric bivariate analysis was performed. If not, the bivariate analysis was done non-parametrically. Yates chi-squared analysis was employed for bivariate analysis of dichotomous variables.

The open-ended queries concerning the use of the HPV-self sampling were subjected to content analysis by two independent observers (SA and EÖ). These two observers came to consensus as to the items that should be included.

Using binomial logistic regression, we computed odds ratios (OR) and 95% confidence intervals (CI) for unadjusted and adjusted models, with the outcome variable being: Could see oneself collecting a sample for HPV testing before the next gynecologic follow-up. Statistica 64 software was used to perform the statistical analysis.

## Results

### Socio-demographic characteristics

As reported in Ref. [44], more than 75% of the participants were 40 years of age or younger. More than half of the participants were married or living with their partner, while slightly fewer than 25% were single (including widowed or divorced). Fewer than one-fourth of the participants had a personal gross annual income below 260 000 Swedish kronor (~ $28 800 USD). Over 75% of the participants were employed, and more than half had finished university education.

### Queries about the HPV self-sampling and other means of follow-up, specific knowledge and logistic issues

The univariate data concerning the HPV self-collected samples, other means of follow-up, as well as specific knowledge and risk assessment show that nearly three fourths of the women could see themselves performing the HPV self-sampling at home prior to their next gynecologic examination (Table 1). The most frequently cited reasons were “saves time/is cost effective” and ease of performance. Concordant with the latter, over 85% of the women endorsed the statement that the self-collection of samples was easy to carry out. On the other hand, the most frequently noted concern regarding the self-sampling was about its reliability.

**Table 1:** HPV self-sampling, other means of follow-up, specific knowledge and risk assessment among the patients with high-grade CIN.

	N	Percentage
Endorses that		
Could see herself doing the HPV self-sampling at home before next gynecologic follow-up		
Yes	353	73.7
No	42	8.8
Did not know	43	9.0
Did not answer	41	8.5
Reasons why the patient would consider doing the HPV self-sampling before her next gynecologic follow-up*		
Saves time/is cost effective	130	27.1
It is easy to perform	115	24.0
Comfort	68	14.2
Can be performed frequently	37	7.7
Can facilitate early detection	32	6.7
Can be done in a relaxed way	22	4.5
Is more readily available	19	4.0
Reasons why the patient would NOT consider doing the HPV self-sampling before her next gynecologic follow-up*		
Concerns about reliability	56	11.7
Concerns about the human factor	34	7.1
Previous CIN diagnosis	6	1.3
Concerns about sending by mail	4	0.8
Endorses that		
HPV self-sampling was easy to carry out		
Yes	411	85.8
No	20	4.2
Only partially	6	1.25
Did not answer	42	8.77
Difficulties with the HPV self-sampling*		
Correct use of applicator	7	1.5
Difficult to carry out	5	1.0
Unclear written instructions	4	0.8
Endorses that		
Received sufficient information for the self-sampling		
Yes	406	84.76
No	12	2.5
Only partially	7	1.5
Did not answer	54	11.27
Information which was missing for the self-sampling*		
Spinning	5	1.0
Drying	3	0.6
Use of the test tube	1	0.2
Handling the urine drop	1	0.2
Breaking the plastic	1	0.2
Confidence in HPV test collected and performed by health professionals to detect cervical cell changes and protect you from cervical cancer, 1 lowest, 10 highest		
9 to 10	336	70.1
7 to 8	53	11.1
5 to 6	10	2.1
3 to 4	2	0.4
1 to 2	1	0.2
Did not answer	77	16.1
Confidence in HPV test from self-collected samples to detect cervical cell changes and protect you from cervical cancer, 1 lowest, 10 highest		
9 to 10	102	21.3
7 to 8	158	33.0
5 to 6	97	20.25
3 to 4	25	5.2
1 to 2	18	3.75
Did not answer	79	16.5
Confidence in Pap smear to detect cervical cell changes and protect you from cervical cancer, 1 lowest, 10 highest		
9 to 10	307	64.1
7 to 8	51	10.6
5 to 6	19	4.0
3 to 4	9	1.9
1 to 2	10	2.1
Did not answer	83	17.3
Specific Knowledge about HPV, CIN and cervical cancer (number of correct answers)		
6	228	47.6
5	108	22.6
4	49	10.2
3	20	4.2
2	17	3.5
1	11	2.3
0	46	9.6
Self-assessed risk of developing cervical cancer without regular gynecologic follow-up, 1 lowest, 10 highest		
10	74	15.5
7 to 9	231	48.2
5 to 6	104	21.7
3 to 4	31	6.5
1 to 2	3	0.6
Did not answer	36	7.5

*More than one option is possible for these queries about the HPV self-sampling

Over 80% of the participants gave high ratings (≥ 7) regarding their confidence in the HPV test collected and performed by health professionals. However, only 54.3% rated the self-collection of samples at that high level. Nearly 75% expressed the high confidence (≥ 7) in the Pap smear.

As also reported in Ref. [44], about 70% of the participants had high specific knowledge, with five or six of the six queries correctly endorsed. Almost 30% answered 4 or fewer of the specific knowledge queries correctly and almost 10% did not correctly answer any of the 6 specific knowledge queries.

Just over 15% of the women considered that without gynecologic follow-up their risk of developing cervical cancer was maximally high (score of 10). Nearly half considered their risk to be high but not maximally (7 to 9), whereas almost 30% viewed their risk as moderate to low.

The total distance travelled had a very wide range, up to 254 kilometers (Table 2). The total travel time needed also varied substantially. Likewise, the costs involved (excluding the examination itself) showed a large range. Detailed cost analyses will be presented in a separate paper. Over half of the women needed to take time away from work in order to attend the gynecologic examination.

**Table 2:** Logistic issues for women with high-grade CIN to attend follow-up gynecologic exam.

Mean	Standard deviation	Minimum	Maximum
Total distance travelled in kilometers			
28.2	28.5	1	254
79 women did not answer			
Total travel time to and from the clinic in minutes, excluding the gynecologic exam itself			
67.3	35.4	10	280
3 women did not answer			
Total cost in Swedish Kronor* excluding the gynecologic exam itself			
497	651	5	8300
89 women did not answer			
Endorses that			
	N	Percentage	
Took time off from work to attend the gynecologic exam			
Yes	254	55.3	
No	205	44.7	
Did not answer	20		
Needed help from another person to attend the gynecologic exam			
Yes	63	13.4	
No	408	86.6	
Did not answer	8		

*One Swedish krona ~ 0.108 US Dollar

### Bivariate findings of note

There was no significant association between readiness to do the self-sampling with age, income or educational level (2-sample “t” test) nor with any of the civil status categories (married, co-living, living apart, single) (Yates chi-squared).

Confidence in HPV tests collected and performed by health professionals and confidence in the HPV test from self-collected samples were correlated (Spearman rho = 0.3, p < 0.0001). Confidence in the Pap smear and confidence in the HPV test collected and performed by health professionals were also correlated (Spearman rho = 0.46, p < 0.0001). However, there was no correlation whatsoever between confidence in the self-sampled HPV test and confidence in Pap smear (Spearman rho = 0.004, p = 0.94). The confidence in the three tests differed significantly (Friedman’s Analysis of Variance (ANOVA) chi-squared = 394, p < 0.0001), with the lowest rank being for confidence in the HPV self-collection.

There was no significant association between readiness to do the self-collection and refraining from another activity or needing another person’s help to attend the exam (Yates chi-squared). Neither total costs nor total time expended were associated with readiness to do the HPV self-sampling (Mann-Whitney test). However, greater total distance traveled was associated with readiness to do the HPV self-sampling (Mann-Whitney test, p = 0.02).

#### Outcome variable: Could see oneself collecting a sample for HPV testing at home before the next gynecologic follow-up

The variable: “Could you see yourself collecting a sample for HPV testing at home before your next gynecologic follow-up” was the outcome measure in the logistic regression analysis. The dichotomization was endorsement versus non-endorsement (negative reply, did not know or did not answer).

In the unadjusted logistic regression model, total distance travelled dichotomized at >35 kilometers, as the independent variable, showed a significant association with readiness to do the HPV self-collection (Table 3). This model included 400 of the study participants. Confidence in the HPV self-collected test was dichotomized at > 6, with the missing data inferentially included as ≤ 6. Thus, this unadjusted model has complete data for the 479 participants. Similarly, since the Specific Knowledge scale score requires correct endorsement of the query, this unadjusted logistic regression analysis includes all 479 participants.

**Table 3:** Logistic regression models for readiness to do HPV self-sampling.

	N	OR	−95% CI	+95% CI	p
Unadjusted Logistic Regression					
Total distance traveled to clinic over 35 km	400	1.77	1.01	3.10	0.038
Confidence in HPV self-sampling (score > 6)	479	8.57	5.20	14.1	<0.0001
Specific knowledge score	479	2.10	1.10	3.8	0.02
Adjusted Logistic Regression Models (for age, annual income, education)*					
Model 1	460				
Specific knowledge score		1.13	1.01	1.26	0.03
Model 2	390				
Total distance traveled to clinic over 35 km		1.94	1.01	3.74	0.047
Confidence in HPV self-sampling (score > 6)		9.14	5.10	16.4	<0.0001
Specific knowledge score		1.21	1.06	1.39	0.005

*The covariates age, annual income and education are non-significant in these adjusted regression models.

The adjusted binomial logistic regression models include age, annual income and education as covariates. Adjusting for these 3 covariates, the Specific Knowledge score retained a significant OR. This multi-variate model included nineteen fewer patients, due to the missing data for age, income and education. Total distance travelled, confidence in the HPV self-collected samples, and specific knowledge score were all in that adjusted model, which included only 390 of the participants.

### Subgroup analyses

We selected two subgroups for further analysis. The first of these is the women who rated their risk as the highest of developing cervical cancer without gynecologic follow-up. The other group includes the women with lower income and education compared to the cohort as a whole. We focus on how these two subgroups of patients view the HPV self-sampling (Table 4). For reference, the left-most column of Table 4 shows the data for the entire group (as per Table 1). Further salient information is described in the text which follows.

**Table 4:** HPV self-collection assessed by patient sub-groups: Those with highest perceived cervical cancer risk and those with lower income and education.

The Entire Group N = 479		Subgroups			
		Highest Perceived Risk 10/10 N = 74		Lower Income & Education < 260 000 Swedish kronor* and without completed university N = 62	
N	Percentage	N	Percentage	N	Percentage
Endorses that					
HPV self-sampling was easy to carry out					
411	85.8%	66	89.2%	56	90.3%
Could see herself doing the HPV self-sampling before next gynecologic follow-up					
353	73.7%	56	75.7%	47	75.8%
Why the patient would consider doing the HPV self-sampling before next gynecologic follow-up**					
Saves time/is cost effective					
130	27.1%	25	33.8%	12	19.4%
It is easy to perform					
115	24.0%	16	21.6%	15	24.2%
Comfort					
68	14.2%	9	12.2%	10	16.1%
Can be performed frequently					
37	7.7%	8	10.8%	2	3.2%
Can facilitate early detection					
32	6.7%	6	8.1%	2	3.2%
Can be done in a relaxed way					
22	4.5%	7	9.5%	4	6.5%
Is more readily available					
19	4.0%	2	2.7%	1	1.6%
Why the patient would NOT consider doing the HPV self-sampling before next gynecologic follow-up**					
Concerns about reliability					
56	11.7%	12	16.2%	7	11.3%
Concerns about the human factor					
34	7.1%	5	6.8%	3	4.8%
Previous CIN diagnosis					
6	1.3%	1	1.4%	0	
Concerns about sending by mail					
4	0.8%	0		0	

*One Swedish krona ~ 0.108 US Dollar

** More than one option is possible for these queries

#### Women with the highest perceived risk

As noted, there were 74 women who indicated that without appropriate gynecologic follow-up they considered their risk to be maximum (10 of 10) of developing cervical cancer. Their mean Specific Knowledge score was higher (5.15 ± 1.2) than for the other participants (2 sample “t” test, p = 0.009). Their confidence in HPV testing by health professionals and in the HPV self-collection was very similar to that of the rest of the participants. However, their mean confidence score for the Pap smear was higher (9.5 ± 1.1) (2 sample “t” test, p = 0.01).

Altogether, 56 of the 74 women who rated their risk to be 10 (75.7%) endorsed that they could see themselves doing the HPV self-sampling, as indicated in the middle column of Table 4. For the remainder of the participants, this percentage was 73.7%. This difference is not statistically significant (Yates chi-squared). Compared to the entire group, a somewhat larger percentage, 33.8% of the patients with the highest perceived risk stated that time and cost effectiveness was a reason for doing the self-collection. Facilitation of early detection, performing the self-collection in a relaxed way and more frequently were also cited slightly more often among this patient subgroup. Nearly 90% of these women considered the self-sampling easy to carry out. Their concerns about reliability were somewhat more frequently cited compared to the patient cohort as a whole.

#### Women with lower income and without university education

Altogether, sixty-two of the participants had an annual personal income below 260 000 Swedish kronor (~ 28 000 USD) and had not completed university education. This subgroup of women had lower mean Specific Knowledge (3.7 ± 2.4, 2 sample “t” test p = 0.00006) compared to the other participants. Their mean perceived risk of developing cervical cancer without gynecologic follow-up was also lower (6.84 ± 2.08, 2 sample “t” test p = 0.01). Their mean confidence in HPV testing by health professionals was similar that of the other women. However, their mean level of confidence in the HPV self-collection was higher (8.0 ± 1.9, 2 sample “t” test p = 0.008). On the other hand, their mean level of confidence in the Pap smears was lower (7.74 ± 2.9, 2 sample “t” test p < 0.0001).

Over 75% of the women in this subgroup were ready to do the HPV self-sampling, as seen in the right-most column of Table 4. Slightly over 90% of these patients considered the self-collection easy to carry out. Their most frequently cited reason (over 24%) for carrying out the self-sampling was ease of performance. Concerns about reliability were slightly less often cited by this subgroup (11.3%), compared to the entire cohort.

## Discussion

The present study provides clear and consistent evidence that the HPV self-sampling is an acceptable option among the vast majority of this cohort of women who have been treated for high grade CIN. Nearly three fourths of the women stated that they would consider performing the self-collection before their next follow-up examination. An even larger percentage of the women considered the procedure easy to perform. The level of confidence which these women indicated in the self-collection, albeit significantly lower than the examinations performed entirely by health professionals (HPV test and Pap smear), was still quite high. Namely, the majority of women ranked their confidence in the HPV self-sampling to be well above the midline. Moreover, confidence in the self-collected samples was the strongest multivariate predictor of readiness to perform the HPV self-collection. Concordantly, the most common reason for not being ready to perform the self-collection was concern about its reliability. This concern is consistent with the somewhat earlier data from the literature [18]. As the efforts to increase the reliability of self-sampling continue to develop [19–22], and, indeed, succeed in eliminating any disparity with clinician-collected samples [23], it is anticipated that confidence in self-collection will also rise among patients such as those in the present cohort.

In the present study, the HPV self-sampling was introduced to the participants as a new way to help prevent cervical cancer. It is possible that this positive presentation encouraged the participants to respond in what they perceived as a socially-desirable manner, notwithstanding the guarantees of confidentiality. However, the finding that confidence in self-sampling was significantly lower than for samples collected by health professionals as well as for Pap smear, suggests that bias due to social desirability is likely to be fairly minimal.

The two other significant multi-variate associations with readiness to perform the HPV self-collection are also noteworthy. First was that the women with higher specific knowledge about HPV, CIN and cervical cancer are those who are the most ready to perform the self-collection. On the other hand, we can identify a group of women with high-grade CIN for whom coming to the clinic exam is likely to be more difficult. Namely, it was those who were obliged to travel longer distances. These women also appear to be more amenable to performing the self-collection, according to the results of the present study. Along these lines, a study of barriers and facilitators to cervical cancer screening among women in rural Ontario, Canada [30] indicates that HPV self-collection was considered a facilitator for screening, and was well accepted in these rural communities.

Our findings that HPV self-collection was well-accepted among the subgroup of women with lower income and lower educational levels are also broadly consistent with the findings that self-collection is a viable, and even preferred option among women who are underscreened [33–35]. As mentioned, women with lower income and education are often at risk for non-attendance to cervical screening programs [5–7]. It is particularly noteworthy that almost all the women in this subgroup considered self-collection of the samples easy to perform.

Another sub-group of patients upon whom we focused attention was those who assessed their own risk of developing cervical cancer without gynecologic follow up as the highest. Although direct data about anxiety were not ascertained in the present study, it can be surmised that these women are the most anxious about their risk of developing cervical cancer. Data from other studies indicate that colposcopy and subsequent HPV testing [47, 48] are associated with substantial distress. The results of the present study suggest that self-collection is a highly acceptable and implementable option among this subgroup of patients who considered their risk to be the highest. Several of the favorable attributes of self-sampling were more often spontaneously noted in this subgroup, namely: facilitation of early detection, the possibility of more frequent performance and that it can be done in a relaxed manner. All of these attributes of self-sampling could conceivably reduce worry and promote greater empowerment among this subgroup of women.

To the best of our knowledge, there have not yet been any published studies explicitly examining the relationship between HPV self-sampling and empowerment. However, a qualitative interview study of young women undergoing HPV DNA and cytological testing indicates that empowerment through the knowledge of results and the possibility to prevent future disease was a key outcome of their participation in the screening program [49]. Moreover, it has been reported that increased empowerment is significantly related to intention to participate in cervical cancer screening programs [50]. On a wider community-based level, empowerment models have shown substantial success in developing coalitions aimed at eliminating disparities in cervical cancer, as well as breast cancer, among African American women in the U.S. [51].

A key component of empowerment which motivates participation in cancer screening is that, in addition to potentially saving one’s life, control is put back into one’s own hands [52]. Concordantly, for patients at high cancer risk or who have been treated for cancer, it has been noted that feelings of abandonment can arise insofar as the patient feels that she has not receive adequate follow-up [53]. As the patient herself becomes more actively involved in decisions about the surveillance strategy, and is better informed about the results, her sense of control through self-management of aftercare is found to improve, with beneficial effects [54, 55]. Within this framework, self-collection for HPV testing would seem to play an important role in empowering women treated for high-grade CIN, and warrants further attention in this light.

A major strength of the present study is the very high participation rate. The efforts of the investigative team in personally explaining the study to all the eligible women seem to have contributed to creating an atmosphere of trust. It has been clearly demonstrated that personal contact encourages women to participate in studies of early cancer detection and prevention [56, 57].

Although the age-adjusted incidence of cervical cancer in Sweden is reportedly similar among women born outside Sweden and those born in the country [58], the cancer mortality rate ratio, adjusted for age at follow-up for the period 1961 to 2009, was significantly higher for women born outside Sweden compared to those who were born in Sweden [16]. Women of ethnic minority backgrounds are generally reported to have lower attendance to cervical cancer screening programs [6, 59–62]. Notably, women who immigrated to Sweden after age 30 reportedly have a low participation in screening [59]. Considering the increased mortality rate ratio among women born outside Sweden and that those who immigrated later are less likely to have Swedish language fluency, outreach is urgently warranted. As noted in Ref. [44], the non-participants in this study, due in all but one case to lack of Swedish language proficiency, were a mean of seven years older than the study participants. Culturally-appropriate intervention programs have been clearly demonstrated to promote screening for cervical cancer and other cancers [63]. Translation of the relevant informational materials into a number of different languages is particularly needed in this regard [46].

It is not known whether the high level of acceptability of self-sampling found among the participants in the present study also holds true for women with high-grade disease who are non-adherent to gynecologic follow-up. Particularly in light of the savings in time and costs, this latter group of patients could certainly benefit from the option of collecting samples at home for HPV testing. Whether they would agree to do so warrants investigation.

Nevertheless, the findings of the present study have broad implications. Notably, even among women who participate in cervical cancer screening programs in Sweden, on-time screening is frequently low [46, 60]. Competing concerns that appear to be more pressing, including having to take time from work, are often noted as impediments to cancer screening among women [46, 57, 64, 65]. In this light, the newly developing guidelines including the potential for acceptable and convenient options such as self-sampling for HPV [1, 4, 9, 11] become particularly promising. These, together with widespread population-based HPV vaccination, that has substantially reduced the need for diagnostic colposcopy [66] hold promise for reduction and eventually even elimination of full-blown cervical cancer. Critical to all these efforts is a well-informed public and an agile, pro-active and fully-informed health care sector.

### Policy implications/follow-up of women treated for high-grade CIN

The wider aim of this study effort was to optimize follow-up management of patients treated for high-grade CIN. Currently, in Sweden, these patients are referred for double testing (HPV and cytology examination) 6 months after treatment within the framework of the organized screening programme. The results from this study show a high level of acceptability of HPV self-sampling among women after treatment for high-grade CIN who adhere to follow-up. Insofar as further research confirms the reliability of HPV self-sampling in comparison to clinician-collected samples, recommendations could be modified. Namely, recommendations could then include the option of self-sampling for women after treatment for high-grade CIN, due to the need for lifelong surveillance of these women related to their increased risk for cervical cancer. Special attention is needed to investigate the acceptability of HPV self-sampling among women treated for high-grade cervical disease who have not adhered to follow-up recommendations.

## Supporting information

S1 File Questionnaire. (DOCX)

S2 File Data set. (XLSX)
