# Supplementary material for: Is self-sampling to test for high-risk papillomavirus an acceptable option among women who have been treated for high-grade cervical intraepithelial neoplasia?
Source: PLoS One. 2018 Jun 18;13(6):e0199038. doi: 10.1371/journal.pone.0199038 (PMC6005489; doi:10.1371/journal.pone.0199038)
Supplement: S1 File — (DOCX) [file pone.0199038.s001.docx]

***English version of the Swedish Questionnaire prepared by the authors***

***using the translation-back-translation method***

**This study concerns knowledge about human papilloma virus (HPV), prevention and about your time and travel-related costs for gynecologic follow-up**

**Part 1. *The following are questions about your background***

*Please mark or fill in the answer to each question that best applies to you*.

| **No.** | **Questions** | | | |
| --- | --- | --- | --- | --- |
| **F101** | How old are you?.............years | | | |
| **F102** | In which municipality do you live? ..................................... | | | |
| **F103** | What is your civil status?  1. Married  2. Cohabiting  3. Have a partner but living separately  4. Single  5. Widowed  6. Other (please specify) | | | |
| **F104** | What is your employment status/main activity? *More than one answer is possible*.  1. Gainfully employed  2. Self-employed  3. Studying  4. Job seeking  5. On sick leave/retired  6. Other (please specify) ……………………………………………………………… | | | |
| **F105** | How many hours per week are you involved in the above activities (including overtime work)? State the time you are working/job seeking/studying etc.) in whole hours ……………… (e.g.:40) | | | |
| **F106** | What is your gross annual income? Income refers to salary, pension, student aid, compensation from insurance fund, income from own business or farm over the entire year. | | | |
|  | - - 1. thousand kronor   2.20-39 thousand kronor  3.40-59 thousand kronor   - - 1. thousand kronor     2. thousand kronor     3. thousand kronor     4. thousand kronor | - - 1. thousand kronor     2. thousand kronor     3. thousand kronor     4. thousand kronor     5. thousand kronor   13.240-259 thousand kronor  14.260-279 thousand kronor | - - 1. thousand kronor   16.300-319 thousand kronor   - 1. - 339 thousand kronor   2. -359 thousand kronor      1. thousand kronor   20.380-399 thousand kronor   - - 1. thousand kronor | - - 1. thousand kronor   23.600-799 thousand kronor  24. 800-999 thousand kronor  25. 1000 thousand or more kronor |
| **F107** | **What is your highest level of completed education? *Only one answer is possible***   \| 1. Nine-year compulsory school 3. University/College  2. High school 4. Other (please specify)………………… \| \| --- \| | | | |

**Part 2. The following are questions about the time and travel related to your follow-up gynecologic examination.**

*Please mark or fill in the answer to each question that best applies to you*.

| **F201** | **Which means of transportation did you use to get here? If more than one, please specify the main one.**   \| 1. By foot 5. Local/regional bus  2. Bicycle 6. Tram/ subway  3. Car as driver 7. Regional train/commuter train  4. Car as passenger 8. Taxi  9. Other, please specify: …………………………………………………………………. \| \| --- \| | | | |
| --- | --- | --- | --- | --- | --- |
| **F202** | **From where did you travel to come to the clinic for the gynecologic examination?**  1. Your home 3. School/Training  2. Your workplace 4. Other (please specify)…………………………………………………………………. | | | |
| **F203** | **Did you do any other errand/business/activity on your way here?**  1. Yes 2. No (**Go directly to F205 )** | | | |
| **F204** | **What was the errand/business/activity which you did on the way here?**  1. Work 6. Visit family and/or friends  2. School 7. Pick up/drop off your child(ren)  3. Shopping 8. Pick up/drop off another person  4. Service (bank, post office, etc.) 9. Other private errand/activity  5. Recreation/leisure activity | | | |
| **F205** | **About how long in minutes did it take you to travel to the clinic? *Exclude the time for any other errands*** …………..minutes | | | |
| **F206** | **What is the approximate distance in kilometers between the place/location from which you traveled and the clinic?** …………. kilometers | | | |
| **F207** | **Where will you go after your visit to the clinic?**  1. Your home 3. School/Training  2. Your workplace 4. Other (please specify)…………………………………………………………………. | | | |
| **F208** | **Which means of transportation will you take to return to your specified location?**  *If more than one, please specify the main one***.**  1. By foot 5. Local/regional bus  2. Bicycle 6. Tram/ subway  3. Car as driver 7. Regional train/commuter train  4. Car as passenger 8. Taxi  9. Other, please specify:…………………………………………………. | | | |
| **F209** | **Approximately how long in minutes do you estimate it will take to travel to your destination after the clinic visit?** …………..minutes | | | |
| **F210** | **Approximately how far in kilometers do you estimate it is to return to your destination after the clinic visit?** …………..kilometers | | | |
| **F211** | *Answer F211-F213 only if you traveled mainly by car***.**  **About how much do you estimate that you paid to travel here? Include the cost per kilometer by car.**  SEK …………….. | | | |
| **F212** | **Did you pay a parking fee?**  1. Yes 2. No **– Go directly to F218** | | | |
| **F213** | **About how much did you pay for parking?**  SEK ……………… | | | |
| **F214** | *Answer F214-F217 only if you traveled mainly by public transportation***.**  **Do you have a travel pass (7 -day pass, 30-day pass, 1-year pass or similar)?**  1. Yes 2. No **– Go directly to F218** | | | |
| **F215** | **How much did your travel pass cost?**  SEK ……………… | | | |
| **F216** | **Did you pay for your trip with a zone ticket (ticket for short or individual trips)?**  1. Yes 2. No **– Go directly to F218** | | | |
| **F217** | **How much did your zone ticket cost?** SEK …………….. | | | |
| **F218** | **Did you need to cancel or postpone some activity in order come to the gynecologic examination**?  1. Yes 2. No **– Go directly to F220** | | | |
| **F219** | What was the activity which you needed to cancel or postpone?  1. Work-related 6. Visit family and/or friends  2. School-related 7. Pick up/drop off child(ren)  3. Shopping 8. Pick up/drop off another person  4. Service (bank, post office, etc.) 9. Other private errand/activity  5. Recreation/leisure activity | | | |
| **F220** | **Did you take time off from work come to the gynecologic examination**?  1. Yes 2. No **– Go directly to F222** | | | |
| **F221** | **How many hours did you take off from work?**  ……………hours | | | |
| **F222** | **Did you need help from another person to be able to come to the gynecologic examination** (for example, child care or other support)?  1. Yes 2. No **– Please go directly to Part 3** | | | |
| **F223** | **What is your relationship to that other person?**  1. Partner 4. Child care provider service-**Go directly to F227**  2. Relative 5. Other (please specify)…………………  3. Friend 999. Prefer not to answer | | | |
| **F224** | **Did that person have to take time off from work?**  1. Yes 2. No 998. Don't know | | | |
| **F225** | **About how many hours do you estimate that this person took off from work?**  ……………hours | | | |
| **F226** | **What is that person’s annual income before taxes?** Income refers to salary, pension, student aid, compensation from insurance fund, income from own business or farm. | | | |
|  | - - 1. thousand kronor   2.20-39 thousand kronor   - - 1. thousand kronor   4. 60-79 thousand kronor   - - 1. thousand kronor   6. 100-119 thousand kronor  7. 120-139 thousand kronor | - - 1. thousand kronor     2. thousand kronor     3. thousand kronor     4. thousand kronor   12. 220-239 thousand kronor  13.240-259 thousand kronor   - - 1. thousand kronor | 15.280-299 thousand kronor   - - 1. thousand kronor   17.320-339 thousand kronor  18. 340-359 thousand kronor  19.360-379 thousand kronor   - - 1. thousand kronor     2. thousand kronor | 22.500-599 thousand kronor  23.600-799  24. 800-999 thousand kronor  25. 1000 or more thousand kronor |
| **F227** | **For approximately how many hours did you need childcare?** ……………hours | | | |
| **F228** | **Do you pay for the childcare service?**  1. Yes 2. No **– Please go directly to Part 3** | | | |
| **F229** | **Approximately how much do you pay per hour for childcare service?**  ……………SEK per hour | | | |

**Part 3. *The following are factual statements about Human papillomavirus (HPV) and related topics***

*Please mark the answer to each question that best applies for you*.

| **No.** | **The following are factual statements about Human papillomavirus (HPV) and related topics. Please indicate whether you were aware of each of these before you participated in this study with the following options: Yes, No or Don’t know. Try to be as honest as possible in your responses.** |
| --- | --- |
| **F301** | **There is a virus which is called Human Papillomavirus (HPV).**  1. Yes 2. No 3. Don't know |
| **F302** | **HPV has various types.**  1. Yes 2. No 3. Don't know |
| **F303** | **HPV is sexually transmitted between partners.**  1. Yes 2. No 3. Don't know |
| **F304** | **Both men and women can be infected with HPV at one or more times in their lives.**  1. Yes 2. No 3. Don't know |
| **F305** | **HPV is most frequently found in young people, but infection can occur in all age groups.**  1. Yes 2. No 3. Don't know |
| **F306** | **Women and men can be infected with HPV without having any symptoms.**  1. Yes 2. No 3. Don't know |
| **F307** | **In most cases, HPV infection clears by itself.**  1. Yes 2. No 3. Don't know |
| **F308** | **A prolonged HPV infection can, in some cases, cause cellular changes in the uterine cervix.**  1. Yes 2. No 3. Don't know |
| **F309** | **Cellular changes over a longer period can lead to cervical cancer.**  1. Yes 2. No 3. Don't know |
| **F310** | **Some types of HPV can lead to other cancers in both women and men.**  1. Yes 2. No 3. Don't know |
| **F311** | **Some types of HPV can lead to genital warts, so-called condyloma.**  1. Yes 2. No 3. Don't know |
| **F312** | **Vaccination is one way to protect oneself against HPV infections that can lead to cell changes and in some cases to cervical cancer.**  1. Yes 2. No 3. Don't know |
| **F313** | **The vaccination is most effective if given before sexual debut.**  1. Yes 2. No 3. Don't know |
| **F314** | **It is important to continue with gynecological check-ups even if one is vaccinated, since vaccination does not provide full protection.**  1. Yes 2. No 3. Don't know |
| **No.** | **The next questions are about your knowledge of HPV before you participated in this study. Mark the answer which best applies to you.** |
| **F315** | **I think I have good knowledge about HPV.**  1. Yes 2. No 3. Don't know |
| **F316** | **If you think that you need more information about HPV, cervical cancer and preventive methods, how would you like to receive that information?**  1. From my midwife or gynecologist/primary care physician  2. Through educational programs on media such as television or radio  3. By health information through work/school  4. Through informational brochures sent by postal mail  5. Via the internet, social media  6. Other (please specify)………………………………. |

**Part 4. The following are some questions about vaccinations against Human Papillomavirus (HPV) and about your risk of developing cervical cancer**

*Please mark or fill in the answer to each question that best applies to you*.

| **No.** | **Questions** | | | | | | | | | | | | | |
| --- | --- | --- | --- | --- | --- | --- | --- | --- | --- | --- | --- | --- | --- | --- |
| **F401** | **Have you been vaccinated against Human Papillomavirus (HPV)?**  1. Yes 2. No **Go directly to F404** 3. Don't know **Go directly to F404** | | | | | | | | | | | | | |
| **F402** | **If yes, what was the vaccination type?**  1. Gardasil 2. Cervarix 3. Don't know | | | | | | | | | | | | | |
| **F403** | **If yes, how old were you when you were vaccinated?**  ………… years old 998. Don't know | | | | | | | | | | | | | |
| **F404** | **On a scale of 10 to 1, where 10 is highest and 1 is lowest, what do you consider your own risk of developing cervical cancer without regular gynecological follow-up?** | | | | | | | | | | | | | |
|  | 10. | 9. | | 8. | 7. | | 6. | 5. | | 4. | 3. | | 2. | 1. |
| **F405** | **How often do you think that you need gynecological follow-up to protect yourself against developing cervical cancer later in life?** | | | | | | | | | | | | | |
|  | 1. Never | | 2. Every year | | | 3. Every other year | | | 4. Every 3^rd^ year | | | 5. Every 4^th^ year or longer | | |

**Part 5. The following are some questions about the self-sampling procedure for HPV**

*Please check the box or write the answer* *which best applies to you for each question*

| **No.** | **Questions** | | | | | | | | | | |
| --- | --- | --- | --- | --- | --- | --- | --- | --- | --- | --- | --- |
| **F501** | **Was the self-sampling procedure easy for you to carry out?**  1. Yes **Go directly to F503** 2. No 3. Only partially | | | | | | | | | | |
| **F502** | **Why wasn’t the procedure easy to carry out or why was it only partially so?**  ………………………………………………………………………………………………………………………………………………  ………………………………………………………………………………………………………………………………………………  ………………………………………………………………………………………………………………………………………………  ………………………………………………………………………………………………………………………………………………  ……………………………………………………………………………………………………………………………………………… | | | | | | | | | | |
| **F503** | **Did you receive enough information about the self-sampling procedure in the instructions?**  1. Yes **Go directly to F505** 2. No 3. Only partially | | | | | | | | | | |
| **F504** | **What information was missing in the instructions for the self-sampling procedure?**  ………………………………………………………………………………………………………………………………………………  ………………………………………………………………………………………………………………………………………………  ………………………………………………………………………………………………………………………………………………  ………………………………………………………………………………………………………………………………………………  ……………………………………………………………………………………………………………………………………………… | | | | | | | | | | |
| **F505** | **On a scale of 10 to 1, where 10 is highest and 1 is lowest, how confident are you that each of the following tests will detect cervical cell changes to thus protect you against cervical cancer:** | | | | | | | | | | |
| 1. **HPV test from a sample collected by health professionals** | | 10. | 9. | 8. | 7. | 6. | 5. | 4. | 3. | 2. | 1. |
| 2. **HPV test from a sample collected by yourself (self-sampling)** | | 10. | 9. | 8. | 7. | 6. | 5. | 4. | 3. | 2. | 1. |
| 3. **Cytology (Pap smear) performed by health professionals** | | 10. | 9. | 8. | 7. | 6. | 5. | 4. | 3. | 2. | 1. |
| **F506** | **Can you see yourself doing the HPV self-sampling again at home before your next gynecological follow-up?**  1. Yes (Go directly to F507) 2. No (Go directly to F508) 3. Don’t know | | | | | | | | | | |
| **F507** | **Please list the reasons why you would consider doing the HPV self-sampling at home**  ………………………………………………………………………………………………………………………………………………  ………………………………………………………………………………………………………………………………………………  ………………………………………………………………………………………………………………………………………………  ………………………………………………………………………………………………………………………………………………  ……………………………………………………………………………………………………………………………………………… | | | | | | | | | | |
| **F508** | **Please list the reasons why you would not consider doing the HPV self-sampling at home**  ………………………………………………………………………………………………………………………………………………  ………………………………………………………………………………………………………………………………………………  ………………………………………………………………………………………………………………………………………………  ………………………………………………………………………………………………………………………………………………  ……………………………………………………………………………………………………………………………………………… | | | | | | | | | | |
